# Mismatch negativity (MMN) amplitude as a biomarker of sensory memory deficit in amnestic mild cognitive impairment

**DOI:** 10.3389/fnagi.2013.00079

**Published:** 2013-11-20

**Authors:** Mónica Lindín, Kenia Correa, Montserrat Zurrón, Fernando Díaz

**Affiliations:** Laboratorio de Psicofisioloxía e Neurociencia Cognitiva, Facultade de Psicoloxía, Universidade de Santiago de CompostelaSantiago de Compostela, Spain

**Keywords:** amnestic mild cognitive impairment, Alzheimer's disease, event-related potentials, mismatch negativity, sensory memory, biomarkers

## Abstract

It has been suggested that changes in some event-related potential (ERP) parameters associated with controlled processing of stimuli could be used as biomarkers of amnestic mild cognitive impairment (aMCI). However, data regarding the suitability of ERP components associated with automatic and involuntary processing of stimuli for this purpose are not conclusive. In the present study, we studied the Mismatch Negativity (MMN) component, a correlate of the automatic detection of changes in the acoustic environment, in healthy adults and adults with aMCI (age range: 50–87 years). An auditory-visual attention-distraction task, in two evaluations separated by an interval of between 18 and 24 months, was used. In both evaluations, the MMN amplitude was significantly smaller in the aMCI adults than in the control adults. In the first evaluation, such differences were observed for the subgroup of adults between 50 and 64 years of age, but not for the subgroup of 65 years and over. In the aMCI adults, the MMN amplitude was significantly smaller in the second evaluation than in the first evaluation, but no significant changes were observed in the control adult group. The MMN amplitude was found to be a sensitive and specific biomarker of aMCI, in both the first and second evaluation.

## Introduction

Mild cognitive impairment (MCI) is a heterogeneous clinical entity characterized by objective evidence of cognitive decline, without any notable impairment in the performance of daily activities. It is also considered as an intermediate stage between the cognitive changes associated with healthy aging and early clinical features of dementia (Petersen, 2004; Winblad et al., 2004). Among the different subtypes of MCI, amnestic MCI (aMCI) is the most likely to progress to Alzheimer's disease (AD) (Petersen et al., 2001, 2009; Petersen, 2004; Winblad et al., 2004; Albert et al., 2011), which is the most prevalent form of dementia in the elderly (Papaliagkas et al., 2009).

The establishment of aMCI biomarkers would be of benefit to clinicians as the biomarkers could be used as objective diagnostic tools, thus allowing early or pre-symptomatic identification of AD, aiding treatment decisions, monitoring disease progress, and providing opportunities for prevention by population screening (Henry et al., 2012). The methods used to search for biomarkers of MCI include neuroimaging techniques (Small et al., 2006; Hämäläinen et al., 2007), cerebrospinal fluid analysis (Perneczky et al., 2011), genetic analysis (Zhang et al., 2012) and electroencephalography (EEG), both quantitative EEG (see Jackson and Snyder, 2008) and event-related potentials (ERPs; see Jackson and Snyder, 2008 and Vecchio and Määttä, 2011).

The use of ERP technique in the search for aMCI biomarkers is founded on three essential characteristics designated as ideal (see Hampel et al., 2010): it is non-invasive, simple to measure and inexpensive. Moreover, the technique has good temporal resolution and allows the study of neurophysiological correlates of sensory-perceptive and pre-attentive processes, the integrity of which are essential for the efficient functioning of higher-level processes and thus the final performance.

Some ERP studies have shown a larger deficit in the controlled processing of information (such as the evaluation of stimuli in working memory) in adults with aMCI than in healthy adults (Golob et al., 2001; Bennys et al., 2007; Missonnier et al., 2007; Lai et al., 2010; Li et al., 2010; Parra et al., 2012). This deficit appears to be more evident in aMCI adults that progress to AD than in those who do not develop AD (Golob et al., 2007; Missonnier et al., 2007). However, studies concerning ERP correlates of involuntary and automatic processing of stimuli in adults with MCI are scarce and the results are inconclusive.

The mismatch negativity (MMN) component is probably the most widely studied ERP component in healthy and clinical populations, in relation to automatic and pre-attentive processing of stimuli. Mismatch negativity was first described for the auditory modality (Näätänen et al., 1978), but has also been reported for other sensory modalities (for a review see Näätänen et al., 2007).

Auditory MMN is a negative wave commonly derived by subtracting the ERP waveform evoked by the standard stimulus from that evoked by the deviant stimulus in passive oddball tasks (which do not require the participant's attention). In young adults, the MMN latency is between 100 and 200 ms, and the amplitude is maximal at frontocentral sites (reversing polarity at mastoid electrodes). In fact, MMN is considered a correlate of pre-attentive processes, which are triggered when the sensory input does not match the echoic memory representation of a prevalent standard stimulus. Therefore, auditory MMN is an objective index of auditory discrimination (automatic detection of changes in the acoustic environment) and an indirect measure of the accuracy of the neural representation of a standard stimulus (see Näätänen and Alho, 1997).

Among the neural generators of MMN, the bilateral supratemporal cortices and predominantly right frontal cortex have been consistently identified (Näätänen et al., 2007). It has been suggested that the supratemporal component is involved in the automatic detection of auditory change and that the frontal component is related to the involuntary attention switch caused by auditory change (Giard et al., 1990; Rinne et al., 2000).

Some studies have shown that the MMN amplitude decreases significantly with age in healthy adults. This has been observed both when the deviant stimulus differs from the standard in duration (Pekkonen et al., 1996; Cooper et al., 2006) or tonal frequency (Czigler et al., 1992; Gaeta et al., 1998; Cooper et al., 2006) and when novel stimuli are presented (Gaeta et al., 1998). It has also been observed with long interstimulus intervals (ISIs) (Czigler et al., 1992; Pekkonen et al., 1996), but not with short ISIs (2.4 s. or less; Pekkonen et al., 1996; Amenedo and Díaz, 1998; Raggi et al., 2013; but also see Czigler et al., 1992; Gaeta et al., 1998; Cooper et al., 2006). In a rather less consistent manner, the latency of MMN also increases with age (Gaeta et al., 1998; Cooper et al., 2006).

Two possible explanations for age-related changes in MMN parameters have been proposed: (i) the sensory memory trace may be poorer or more degraded in older than in younger subjects, reflecting an inaccurate representation of standard stimuli by the brain, and/or (ii) a deficient comparator mechanism fails to detect a mismatch between the representation of the standard and the deviant stimuli (Gaeta et al., 1998).

In studies with AD patients, changes in MMN have been observed under some task conditions. For ISIs of 1.3 s or less, no significant differences between control adults and adults with AD were observed in the MMN amplitude elicited by changes in tonal frequency (Pekkonen et al., 1994; Kazmerski et al., 1997; Gaeta et al., 1999; Brønnick 2010) or by presentation of novel stimuli (Kazmerski et al., 1997; Gaeta et al., 1999). However, in AD patients the MMN amplitude was significantly smaller for an ISI of 3 s than for the shorter ISI condition (1 s), while in control adults the MMN amplitude was stable across both ISIs (Pekkonen et al., 1994). These results suggest that sensory memory trace decays faster in AD patients than in healthy controls, although auditory discrimination was not affected (Pekkonen et al., 1994).

In the only published study to date evaluating the effect of MCI on MMN parameters, Mowszowski et al. (2012) recorded ERPs in a sample of 14 healthy adults and 28 adults with MCI, in a passive oddball task in which the standard and deviant stimuli differed in duration (standard: 50 ms, deviant: 100 ms). In the MCI group, all participants showed impairment in several cognitive domains. Of these, half were amnestic subtype (aMCI) Multiple Domain and half non-amnestic subtype (naMCI) Multiple Domain. The authors found no differences between the MCI and control groups in MMN amplitude or latency, or between the aMCI and naMCI subtypes at frontocentral locations. However, they did observe that at mastoid locations, the MMN amplitude was smaller in the MCI group than in the control group, which the authors considered reflect of the inefficiency of processing information in an early pre-attentional stage in the MCI group.

The recent study by Mowszowski et al. (2012) provided some interesting results, but also presented some limitations. Thus, the differences in MMN amplitude between MCI and control adults were obtained at mastoid electrodes, but not at the frontocentral locations, where MMN is typically identified and analyzed. Moreover, the analysis did not take into account the possible effects of interactions between the Age and Group factors, although previous studies have reported age effects on MMN amplitude to changes of stimuli duration (Pekkonen et al., 1996; Gaeta et al., 1998; Cooper et al., 2006). Moreover, the MCI group was heterogeneous, as it included both amnestic and non-amnestic multidomain MCI patients.

The aims of the present study were as follows: (1) to determine any differences in MMN parameters between healthy adults and adults with aMCI; (2) to evaluate whether such differences between healthy adults and adults with aMCI are affected by age, by considering two age subgroups (50–64 years and 65 years and over); (3) to determine whether the differences in MMN parameters between healthy and aMCI adults are maintained in a second evaluation, conducted 18–24 months after the first evaluation, and (4) to evaluate whether MMN changes associated with aMCI are sensitive and specific biomarkers of this syndrome.

We used an auditory-visual attention-distraction task [based on the task designed by Escera et al. (1998), see Methods section]. This task was presented to a sample of healthy control adults and adults with aMCI during two evaluations separated by an interval of between 18 and 24 months. Auditory MMN was obtained by subtracting the ERP waveform evoked by standard stimuli from the waveform evoked by the deviant or novel stimuli (deviant *minus* standard, and novel *minus* standard).

To our knowledge, this is the first study designed to determine whether MMN parameters (amplitude and/or latency) are sensitive and specific biomarkers of aMCI, whether their effectiveness in identifying adults with this syndrome interacts with age, and also whether the MMN parameters change over time in healthy control and aMCI adults.

## Materials and methods

### Participants

Participants were 56 healthy adult volunteers (35 women, 21 men; age range: 50–87 years old; mean = 65.7 years, *SD* = 9.1), recruited from Primary Care Health Centres in Santiago de Compostela and Vigo (Galicia, Spain) and referred to our research group by their general practitioners (GPs). The participants had no history of clinical stroke, traumatic brain injury, motor-sensory defects, or alcohol or drug abuse/dependence, and they were not diagnosed with any significant medical or psychiatric illnesses. To control for the effects of depression, adults with a score of more than 10 in depression screening (Geriatric Depression Scale, GDS; Yesavage et al., 1983) were excluded from the study. All participants had normal audition and normal or corrected-to-normal vision. Most of them were right-handed, as assessed by the Edinburgh inventory (Oldfield, 1971), except for one left-handed and one ambidextrous participant.

After giving their written informed consent, participants were referred by their GPs to the Psychogerontology Group (see Juncos-Rabadán et al., 2013) with whom we participate in a collaborative research project. They conducted the neuropsychological evaluation and the clinical diagnosis of MCI. The participants were then referred to our laboratory for psychophysiological (ERP) evaluation.

The participants provided us with information about years of education, as well as socioeconomic, medical and personal data, received extensive psychological and neuropsychological evaluations, and were diagnosed and classified as control or aMCI. They underwent the following tests: (1) The Mini-Mental State Examination (MMSE, Folstein et al., 1975; Spanish version, MEC by Lobo et al., 1999), which assesses general cognitive functioning; (2) The Californian Verbal Learning Test [CVLT, Delis et al., 1987; Spanish version, TAVEC by Benedet and Alejandre (1998)], which assesses short-delay free recall, short-delay recall with semantic cues, and long-delay free recall; (3) The Spanish version of the Cambridge Cognitive Examination (CAMCOG-R), which assesses deterioration in specific domains such as language, attention-calculation, praxis, perception, and executive functioning (Huppert et al., 1996) and is sensitive to MCI detection (Gallagher et al., 2010); and (4) The Spanish version of the vocabulary test of the Wechsler Adult Intelligence Scale (WAIS, Wechsler, 1988).

Two evaluations, separated by an interval of between 18 and 24 months, were carried out. In the first evaluation, the Control group (CG) comprised 30 adults aged between 50 and 84 years (mean: 63.9 years, *SD*: 8.4) with normal cognitive and memory functioning, and the aMCI group comprised 26 adults aged between 51 and 87 years (mean: 67.8 years, *SD*: 9.3). In the second evaluation, only 27 (18 control and 9 aMCI) of the initial sample of 56 participants agreed to participate in the ERP recordings. Two of the 18 control adults and 1 of the 9 aMCI adults were excluded because of excessive artifacts in their recording, and 1 aMCI adult died. Nine of the 16 control adults with valid recordings were selected with the aim of matching their ages with the aMCI group. Consequently, the final sample in the second evaluation consisted of 16 adults: 9 control adults (range age: 59–73 years) and 7 aMCI adults (range age: 62–89 years), whose diagnosis was maintained between evaluations.

In both evaluations, control adults scored higher than the cut-off on memory, general cognitive functioning, and specific cognitive domain tests. The aMCI subjects met the general criteria for MCI outlined by Albert et al. (2011) and the criteria for aMCI proposed by Petersen and colleagues (Petersen, 2004; Dubois et al., 2007).

The aMCI adults fulfilled the following criteria: (1) memory complaints corroborated by an informant; (2) performance of less than 1.5 *SD*s below age norms for the CVLT; (3) no significant impact on daily living activities; and (4) without dementia, according to the National Institute of Neurological and Communicative Diseases and Stroke/Alzheimer's Disease and Related Disorders Association (NINCDS-ADRDA) and the Diagnostic and Statistical Manual of Mental Disorders (DMS-IV) criteria. The Lawton and Brody Index (Lawton and Brody, 1969) was used to evaluate instrumental activities of daily living (IADL). Nine of the aMCI adults fulfilled criteria for multiple domain amnestic MCI (mda-MCI), and 17 subjects fulfilled criteria for single domain amnestic MCI (sda-MCI). With respect to general cognitive functioning, the mda-MCI subjects scored less than 1.5 *SD*s below age- and education-related norms in the MEC and in at least two cognitive subscales of the CAMCOG-R. For the analysis of the present study, the two subgroups of aMCI were not differentiated, because they did not show any differences in MMN parameters (see Results section), and they were consequently regrouped into a single group of adults with aMCI.

The research project was approved by the Galician Clinical Research Ethics Committee (CEIC) and performed in accordance with the ethical standards established in the 1964 Declaration of Helsinki (Lynöe et al., 1991).

### Stimuli and task

An auditory-visual attention-distraction task adapted from Escera et al. (1998, 2001) was used. This included a passive auditory oddball task and an active Go/NoGo three-stimuli visual oddball task. Participants were presented with 500 auditory-visual (A-V) stimuli pairs (divided in two blocks separated by a 2-min rest interval). Each pair included an auditory stimulus (150 ms duration) followed by a visual stimulus (200 ms duration), with an interval of 300 ms (onset-to-onset) between them, and a 2-s interval between each pair. Participants were asked to attend to the visual stimuli and ignore the auditory stimuli.

Auditory stimuli were sounds, presented binaurally via headphones, with 75 dB SPL intensity. Three kinds of sounds were presented: 70% were standard stimuli (tone bursts, 1000 Hz), 15% were deviant stimuli (tone bursts, 2000 Hz), and 15% were novel stimuli (different each time: glass crashing, ringing, etc …). Visual stimuli were numbers (2, 4, 6, or 8), letters (a, e, c, or u) or triangles (pointing up, down, right, or left). Participants were asked to respond to the numbers (33%) with one hand and to the letters (33%) with the other hand, by pressing a different button in each case (Go condition), and they were asked to inhibit their responses to triangles (34%, NoGo condition). Response buttons were counterbalanced among participants. For this task, each participant underwent two evaluations separated by an interval of between 18 and 24 months.

### Electroencephalographic (EEG) recording

The participants were seated on a comfortable chair in a Faraday chamber, with attenuated levels of light and noise, and were instructed to move as little as possible during the recording. Visual stimuli were presented with a subtended visual angle of 1.7° × 3.3° of arc, on a 19″ flat screen monitor with a vertical refresh rate of 120 Hz. The monitor was located one meter away from the participant. The electroencephalogram was recorded from 49 ring electrodes placed in an elastic cap (Easycap, GmbH), according to the International 10-10 system. All electrodes were referenced to an electrode attached to the tip of the nose, and an electrode positioned at Fpz served as ground. The horizontal electro-oculogram (EOG) was recorded from two electrodes placed at the outer canthi of both eyes, and the vertical EOG was recorded from two electrodes placed supra and infra-orbitally on the right eye. The EEG was continuously digitized at a rate of 500 Hz (bandpass 0.01–100 Hz), and electrode impedances were kept below 10 kΩ.

Once the signal was stored, ocular artifacts were corrected and the EEG was then segmented by extraction of -100 to 650 ms epochs, synchronized with each auditory stimuli. These were then classified *a posteriori* as Standard, Deviant and Novel, depending on the type of auditory stimulus. The signal was passed through a digital 0.1–30 Hz (24 dB/octave slope) bandpass filter and epochs were corrected to the mean voltage of the 100-ms pre-stimulus recoding period. EEG segments exceeding ±100 μV and the first five epochs of each block were automatically excluded from the averages. Finally, to identify and measure MMN, we obtained the deviant *minus* standard (D-S) and novel *minus* standard (N-S) difference waveforms.

### Data analyses

**Figure 1:**
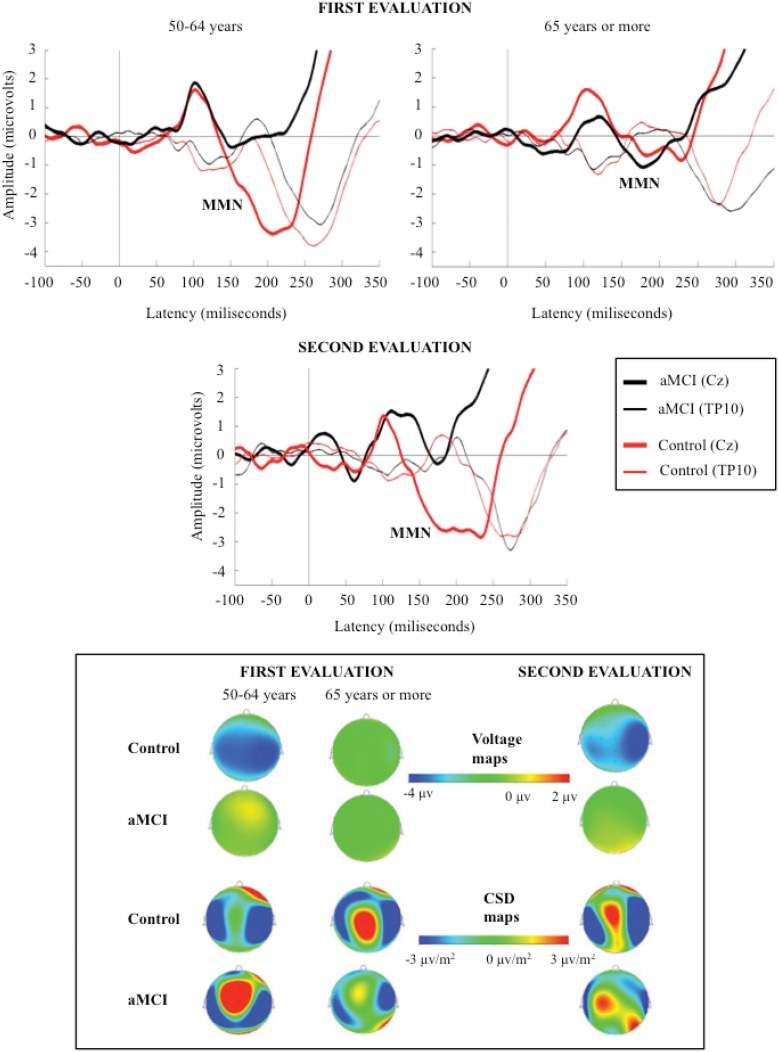
**Grand-average event-related potentials waveforms, measured at Cz (top), during the 350 ms after the stimulus, and voltage and current source density (CSD) maps for MMN maximum peak (bottom), in the novel minus standard difference waveforms, for control and aMCI adults, in the first (for the two age subgroups) and second evaluations**.

The MMN component was identified as a negative wave in the 125–260 ms interval, and it was evaluated at the Cz electrode site (where the amplitude was maximal). The polarity of the component was reversed at temporal (TP9 and TP10) electrode sites (see Figure 1).

In the first evaluation, the MMN component was identified in the N-S and D-S difference waveforms, for the two groups of participants (CG and aMCI group). In the second evaluation, MMN was also identified in the N-S and D-S difference traces for the control adults; however, for the aMCI adults, MMN was only identified in the N-S difference trace and it was absent in the D-S difference trace.

The MMN amplitude (in microvolts, from the maximum peak to the baseline) and latency (in milliseconds, from the auditory stimulus onset to the maximum peak) were measured. Current source density (CSD) and voltage maps were also obtained for topographic analysis.

### Statistical analyses

In the first evaluation, one-factor analysis of variance (ANOVA) was used to investigate the effect of the Group factor on the MMN amplitude and latency (measured at Cz) in the N-S and D-S difference waveforms. The analysis considered a dependent variable (MMN amplitude or latency) and an independent factor (Group, three levels: CG, sda-MCI, and mda-MCI groups). As there were no significant differences between aMCI subgroups for MMN parameters, both subgroups were regrouped as a single aMCI group for the following analysis.

Two-factor ANOVAs was used to investigate the effect of Group and Age factors on the MMN amplitude and latency (measured at Cz) in the N-S and D-S waveforms. The analysis included a dependent variable (MMN amplitude or latency) and two independent factors, Group (two levels: CG, and aMCI) and Age (two levels: middle-aged adults: 50–64 years, and older adults: 65 years and over).

In the second evaluation, a one-factor ANOVAs (Group) was used to investigate the Group factor effect on the MMN amplitude and latency (measured at Cz) in the N-S difference waveforms. The Age factor was not evaluated because of the small number of participants (9 in the CG and 7 in the aMCI group). In this evaluation, the effect of the Group factor on MMN in the D-S difference was not evaluated because no MMN component was observed in this difference waveform in aMCI adults.

Finally, *t*-tests for related samples were used to evaluate the changes in the MMN parameters (in the N-S difference waveform) between the first and the second evaluation, in each group of participants (9 control adults and 7 aMCI adults).

When the ANOVAs revealed significant factor and interactions effects, further comparisons of the mean values were carried out by paired multiple comparisons (adjusted to Bonferroni correction). Differences were considered significant at *p* < 0.05.

The receiver-operating characteristics (ROC) method was used to assess the capacity of MMN parameters to discriminate aMCI adults from control adults. A line diagram was constructed with the sensitivity (true positive rate) plotted on the vertical axis and the false positive rate (1 minus specificity) on the horizontal axis. The ROC curve was constructed by finding the sensitivity and specificity for a range of values of the continuous variable (MMN parameters). The tests were considered to be ideal when the area under the curve (AUC) was higher than 0.7.

All statistical analyses were performed with IBM SPSS Statistics package v.19 for Windows.

## Results

### Demographic and neuropsychological data

In both evaluations, the groups were matched according to age and level of education. The demographic and neuropsychological measurements are summarized in Table 1, together with the between-group differences calculated by the corresponding analysis. For an extensive description of the global samples, the inclusion/exclusion criteria, the tests used, and the diagnosis and classification criteria, see Juncos-Rabadán et al. (2013).

**Table 1:** **Mean values and standard deviations (in parentheses) of the demographic and neuropsychological measures, for control and amnestic MCI (aMCI) adults**.

	**Control**	**aMCI**	***p*<**	
**FIRST EVALUATION**	***N* = 30**	***N* = 26**		
Age	63.9 (8.4)	67.8 (9.3)	NS	
Years of education	9.4 (4.4)	10.15 (4.7)	NS
Gender (F/M)	21/9	14/12	
WAIS, vocabulary	49.9 (11.7)	46.7 (13.4)	NS
MMSE	28.5 (0.9)	25.8 (2.1)	0.001 Control > aMCI
CVLT (short-delay free recall)	10.2 (2.3)	3.9 (1.8)	0.001 Control > aMCI
CVLT (short-delay cued recall)	12 (2.3)	5.8 (2.2)	0.001 Control > aMCI
CVLT (long-delay free recall)	11.4 (2.2)	4.5 (3)	0.001 Control > aMCI
Depression (GDS)	2.9 (2.1)	3.8 (3.1)	NS
**SECOND EVALUATION**	***N* = 9**	***N* = 7**		
Age	66.6 (5.5)	74.4 (10.1)	NS	
Years of education	8.2 (4.3)	11.1 (4.5)	NS	
Gender (F/M)	(7/2)	(3/4)		
WAIS, vocabulary	46.4 (9)	49 (16.7)	NS	
MMSE	28.1 (1.2)	23.3 (5.1)	0.01	Control > aMCI
CVLT (short-delay free recall)	11.6 (2.9)	2.7 (2.6)	0.001	Control > aMCI
CVLT (short-delay cued recall)	13 (1.9)	5 (2.5)	0.001	Control > aMCI
CVLT (long-delay free recall)	12.3 (3.3)	4.7 (3.6)	0.001	Control > aMCI
Depression (GDS)	2.2 (0.9)	2.7 (1.1)	NS	

The ANOVA results for the Group factor are also shown.

### ERPS

The factors under consideration did not have any significant effects on the MMN latency or amplitude in the D-S difference waveforms. Therefore, we will report only the results obtained for MMN in the N-S difference waveforms, for both the first and the second evaluation.

The mean MMN amplitudes (μV) and latencies (ms) obtained in both evaluations are shown in Table 2. The grand average ERP waveforms of the N-S difference traces and the voltage and CSD maps for MMN maximum amplitude peaks in the first and the second evaluations are shown in Figure 1. Voltage maps for MMN revealed larger amplitudes for the CG than for the aMCI group. In both groups, the CSD maps showed bilateral sinks at temporoparietal scalp regions and a right frontal source. However, between-group topographical differences in CSD maps were also observed. During the first evaluation, a widespread centroparietal source was observed in control adults in the older age subgroup (65 years and over) and in aMCI adults in the middle-aged subgroup (50–64 years); however, this source was not observed in the middle-aged control adults or in the older adults with aMCI.

**Table 2:** **Mean values and standard deviations (in parentheses) of the auditory MMN amplitudes (in μV) and latencies (in ms), measured at the Cz electrode, in the novel minus standard (N-S) and deviant minus standard (D-S) difference waveforms, for the two diagnostic groups (control and amnestic MCI adults)**.

**Group**	**Age**	**N-S MMN**		**D-S MMN**	
		**Amplitude**	**Latency**	**Amplitude**	**Latency**
**FIRST EVALUATION**					
Control adults (*N* = 30)	50–64 (*N* = 15) (M: 56.8 years, *SD*: 3.9)	−5.3 (3.3)	207 (27)	−1.9 (2.0)	228 (25)
	≥65 (*N* = 15) (M: 71.1 years, *SD*: 4.9)	−2.6 (2.2)	202 (33)	−1.1 (1.5)	207 (32)
aMCI adults (*N* = 26)	50–64 (*N* = 10) (M: 58.8 years, *SD*: 4.3)	−2.1 (2.8)	187 (35)	−1.2 (1.2)	208 (27)
	≥65 (*N* = 16) (M: 73.5 years, *SD*: 6.7)	−2.5 (2.2)	186 (34)	−2.2 (2.1)	201 (33)
**SECOND EVALUATION**					
Control adults (*N* = 9)	59–73 (M: 66.6 years, *SD*: 5.5)	−4.7 (3.5)	205 (56)	−1.9 (1.2)	210 (35)
aMCI adults (*N* = 7)	62–89 (M: 74.4 years, *SD*: 10.1)	−1.1 (2.0)	192 (34)	–	–

In the first evaluation, the data for the two age subgroups (50–64 years and 65 years and over) in each group (control and aMCI) are shown.

M: mean, SD: standard deviations.

#### First evaluation

For MMN latency, the two-factor ANOVA (Group × Age) showed significant effects of the Group factor [*F*_(1, 52)_ = 4.1, *p* < 0.048], as latency was significantly shorter in the aMCI group than in the CG. For MMN amplitude, the two-factor ANOVA revealed significant effects of the Group factor [*F*_(1, 52)_ = 5.4, *p* < 0.023] and the Group × Age interaction [*F*_(1, 52)_ = 4.9, *p* < 0.031], because the amplitude was significantly larger (*p* < 0.004) in the CG than in the aMCI group, only for the middle-aged subgroup (50–64 years). Furthermore, for the CG, the amplitude was significantly larger (*p* < 0.008) in the middle-aged than in the older adults.

#### Second evaluation

For MMN latency, the one-factor ANOVA revealed no significant factor (Group) effect. For MMN amplitude, the ANOVA showed significant effect of the Group factor [*F*_(1, 14)_ = 5.66, *p* < 0.032], as the amplitude was significantly larger for the CG than for the aMCI group.

#### First vs. second evaluation

**Figure 2:**
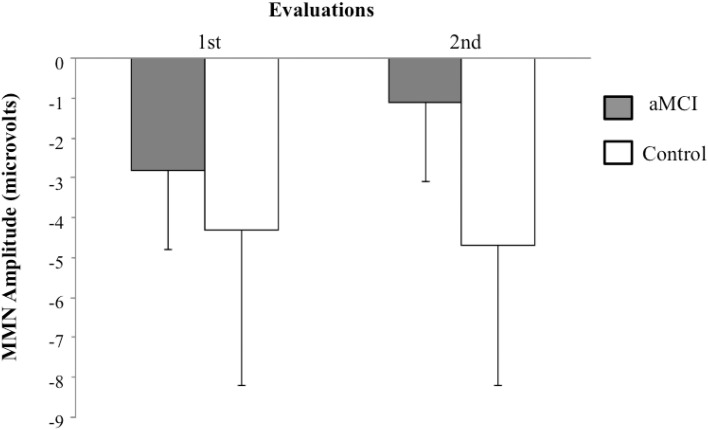
**Means and standard deviations for MMN amplitudes (in μV) in the novel minus standard difference, in the first and the second evaluations, for control (*N* = 9) and aMCI (*N* = 7) adults**.

The *t*-test for related samples did not show any significant differences for the MMN latency between the first and second evaluations. However, for the MMN amplitude, the analysis revealed significant differences within the aMCI group, as the MMN amplitude was significantly smaller in the second evaluation (−1.1 μV) than in the first evaluation (−2.8 μV) [*t*_(6)_ = −2.9, *p* < 0.027]. The MMN amplitude and latency in the control group did not differ significantly between the first and second evaluations (see Figure 2).

### Sensitivity and specificity

**Figure 3:**
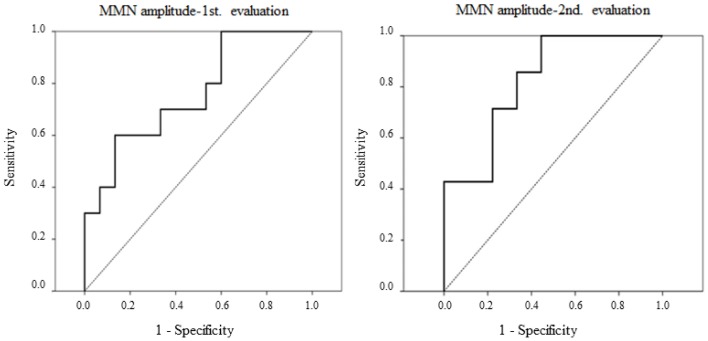
**Receiver-operating characteristics (ROC) curves are represented for MMN amplitudes in novel minus standard difference, in the first and the second evaluations, for the aMCI and control adults comparison. Left:** MMN amplitude for the middle-aged subgroup in the first evaluation. **Right:** MMN amplitude in the second evaluation.

The ROC curves for MMN amplitudes (N-S difference waveforms), in the first and second evaluations, for the aMCI group and CG comparisons are shown in Figure 3.

In the first evaluation, MMN amplitude showed 0.7 sensitivity and 0.66 specificity (AUC = 0.76) for the discrimination between aMCI and control middle-aged adults. However, the MMN latency showed very low sensitivity and specificity (AUC = 0.34). In the second evaluation, the MMN amplitude was also sensitive and specific in discrimination between aMCI and control groups, with 0.71 sensitivity and 0.78 specificity (AUC = 0.82).

## Discussion

Mismatch negativity (MMN) component was identified in both groups of participants (control and aMCI). As expected, CSD maps showed bilateral sinks at temporal scalp regions and a right frontal source for this component, presumably reflecting the MMN generator activity, located in the auditory supratemporal cortices and the frontal cortex, respectively (see Näätänen et al., 2007).

Control and aMCI adults showed differences for MMN component of the N-S difference waveforms in two evaluations separated by an interval of between 18 and 24 months. In the first evaluation, the MMN amplitude was significantly smaller in the aMCI adults than in the control adults, though only for the middle-aged subgroup (between 50 and 64 years of age). The MMN latency was significantly shorter in the aMCI group than in the CG. In the second evaluation, the MMN amplitude was also significantly smaller in the aMCI group than in the CG, and the aMCI group showed a decrease in MMN amplitude from the first to the second evaluation, whereas the CG did not show significant changes between evaluations.

### MMN latency

In the first evaluation, the MMN latency was significantly shorter in the aMCI group than in the CG. In the second evaluation, although the mean values and SD for both groups were similar to those of the first evaluation, the Group factor was not statistically significant, probably because the sample size was smaller in the second evaluation. This result is intriguing because no significant differences in MMN latencies were observed in other MMN studies comparing AD patients and healthy control subjects (Kazmerski et al., 1997; Gaeta et al., 1999; Brønnick 2010) or in the only previous study comparing a MCI group with a control group (Mowszowski et al., 2012).

Although the shorter MMN latency in the aMCI group than in the CG in the first evaluation of this study must be considered with caution. Interestingly, Mowszowski et al. (2012) also observed slightly shorter (but non-significant) MMN latencies (measured at Fz and Cz electrodes) in the MCI than in the control group (see Table 2, page 214, of the cited study). We tentatively speculate that in participants with aMCI, earlier closure of the comparison (in echoic memory) of each novel stimulus with the stored model of a standard stimulus may occur, resulting in a mismatch. This early closure may be premature and related to the deterioration of echoic memory. However, this hypothesis must be addressed in greater detail in future studies.

### MMN amplitude

In the first evaluation, the MMN amplitude was significantly larger in the CG than the aMCI group, only for the middle-aged subgroup (50–64 years). This may indicate some impairment, in the middle-age aMCI adults, of the automatic detection mechanism of disparities when the novel stimuli are presented. This mechanism depends on maintaining an echoic memory trace of the standard stimulus, with which it automatically compares each novel (or deviant) stimuli presented. Näätänen et al. (2011), (2012) suggested that MMN deficits may be at least partly explained by dysfunction of the N-methyl-D-aspartate (NMDA) receptor system, which usually binds to the neurotransmitter glutamate. Consequently, reduced MMN in aMCI adults may signify a more general functional deficiency involving glutamatergic dysfunction, as suggested by Mowszowski et al. (2012).

The ROC analysis for the first evaluation showed that MMN amplitude could be considered a biomarker of aMCI in middle-aged adults, discriminating between the two groups with a sensitivity of 0.70 and specificity of 0.66 (AUC = 0.76, in ROC curves).

For adults 65 years old or more, there were no differences in MMN amplitude between the control and aMCI groups. This is probably due to a significant age-related decrease in MMN amplitude in the CG, as also found by Gaeta et al. (1998). For the aMCI group, the MMN amplitude did not differ between the two age subgroups. Thus, in the older adults (65 years and over), the lack of differences between the CG and the aMCI group may be due to an age-related decline in the mechanism for echoic memory trace maintenance and/or the pre-attentional mechanisms involved in the automatic detection of differences in the acoustic environment, which may mask the effects of aMCI on that parameter.

The CSD maps for MMN showed similar sources and sinks in control and aMCI adults. These maps also revealed differences in the first evaluation, which are consistent with the results obtained for the MMN parameters in the present work. Thus, they revealed a centroparietal source in middle-aged adults (50–64 years) with aMCI, but not in the middle-aged control adults, which is consistent with the significant between-group differences (control and aMCI adults) for MMN amplitude. Moreover, in accordance with the observed effects of aging on the MMN amplitude in the control group, this source was also observed in the older control adults (65 years and over). Within the framework of the Scaffolding Theory of Aging and Cognition (STAC) proposed by Park and Reuter-Lorenz (2009), we believe that the said source may reflect a brain that is adapting, through neural scaffolding, to the functional and structural changes that appear, either due to aMCI in middle–aged adults or to healthy aging.

The present ERP results are partly consistent with those obtained by Mowszowski et al. (2012) for the MMN amplitude evoked by deviant stimuli, which differed from the standard stimuli in duration (100 and 50 ms, respectively). These authors observed a larger MMN amplitude in healthy adults than in a multi-domain MCI group (for an age range of 50–90 years in both groups), although only at mastoid locations, where MMN shows polarity reversal, but not at the frontocentral locations, where the amplitude of this negative component is maximal.

Our results are also consistent with those reported by Pekkonen et al. (1994) for AD patients and healthy controls. These authors observed a significant decrease in MMN amplitude, in the AD group, from an ISI of 1 s to an ISI of 3 s, which they interpreted as a weakening of echoic memory trace with increasing ISI in people with AD. In the present study, the interval between auditory stimuli was 2.35 s. We also presented visual stimuli between the auditory stimuli, which required attention and often response. The combination of both factors may have affected the maintenance of echoic memory trace of standard auditory stimuli in aMCI participants, but not in CG participants.

In the second evaluation (conducted between 18 and 24 months after the first), comparison of the MMN parameters in a subsample of participants again showed a significantly larger MMN amplitude in control adults than in adults with aMCI. As found in the first evaluation, the MMN amplitude may be considered a biomarker of aMCI, discriminating between the two groups with 0.71 sensitivity and 0.78 specificity (AUC = 0.82, in ROC curves). Moreover, the characteristics of the MMN component make it an ideal biomarker: it is an automatic ERP component, which is not dependent on the attention given by the subject to the task and, moreover, it is obtained in a non-invasive manner and is simple and inexpensive to measure.

The MMN amplitude was also significantly smaller in the second evaluation than in the first in the group with aMCI, while it did not differ between evaluations in the CG. This may indicate a progressive deterioration, in aMCI adults, of the neural mechanisms involved in the maintenance of sensory trace and/or the pre-attentive mechanisms for automatic detection of changes in the acoustic environmental. The findings highlight the importance of longitudinal studies in determining the evolution of deficits detected in a first assessment in participants with aMCI, as well as the diagnostic and prognostic value of psychophysiological markers.

This study is not without limitations, mainly due to the small sample size in the second evaluation. Future studies, with a large sample of participants in all follow-up evaluations, should (1) confirm the effect of the interaction between age and diagnostic group on MMN amplitude, and (2) determine any differences in how the MMN amplitude decreases over time in adults with MCI who progress to AD and in adults with MCI who do not develop AD.

Despite these limitations, the present study showed that the MMN amplitude was smaller in adults with aMCI than in control adults (in the middle-aged subgroup in the first evaluation and in the whole sample in the second evaluation, conducted between 18 and 24 months after the first). In addition, in participants with aMCI, the MMN amplitude was smaller in the second evaluation than in the first, whereas no difference was observed in the control group. The results of this study suggest that MMN amplitude can be a fairly sensitive and specific psychophysiological biomarker for the identification of adults with aMCI.

### Conflict of interest statement

The authors declare that the research was conducted in the absence of any commercial or financial relationships that could be construed as a potential conflict of interest.
